# Sufficient water intake maintains the gut microbiota and immune homeostasis and promotes pathogen elimination

**DOI:** 10.1016/j.isci.2024.109903

**Published:** 2024-05-03

**Authors:** Kensuke Sato, Mariko Hara-Chikuma, Masato Yasui, Joe Inoue, Yun-Gi Kim

**Affiliations:** 1 Department of Microbiology, School of Pharmacy, Kitasato University, Tokyo 108-8641, Japan; 2 Research Center for Drug Discovery, Faculty of Pharmacy and Graduate School of Pharmaceutical Sciences, Keio University, Tokyo 105-8512, Japan; 3 Institute for Advanced Biosciences, Keio University, Yamagata 997-0052, Japan; 4 Systems Biology Program, Graduate School of Media and Governance, Keio University, Fujisawa 252-0882, Japan; 5 Department of Pharmacology, Keio University School of Medicine, Tokyo 160-8582, Japan; 6 Division of Biochemistry, Faculty of Pharmacy and Graduate School of Pharmaceutical Sciences, Keio University, Tokyo 105-8512, Japan

**Keywords:** Biological sciences, Immunology, Microbiology, Microbiome

## Abstract

Water is the most abundant substance in the human body and plays a pivotal role in various bodily functions. While underhydration is associated with the incidence of certain diseases, the specific role of water in gut function remains largely unexplored. Here, we show that water restriction disrupts gut homeostasis, which is accompanied by a bloom of gut microbes and decreased numbers of immune cells, especially T_h_17 cells, within the colon. These microbial and immunological changes in the gut are associated with an impaired ability to eliminate the enteric pathogen *Citrobacter rodentium*. Moreover, aquaporin 3, a water channel protein, is required for the maintenance of T_h_17 cell function and differentiation. Taken together, adequate water intake is critical for maintaining bacterial and immunological homeostasis in the gut, thereby enhancing host defenses against enteric pathogens.

## Highlights

• Water restriction induces constipation without dehydration
• Water restriction changes the number and the composition of gut microbiota
• Water restriction reduces colonic T_h_17 cells and impairs clearance of *C. rodentium*
• AQP3, a water channel protein, is required for the maintenance of colonic T_h_17

## Graphical abstract

**Figure:**
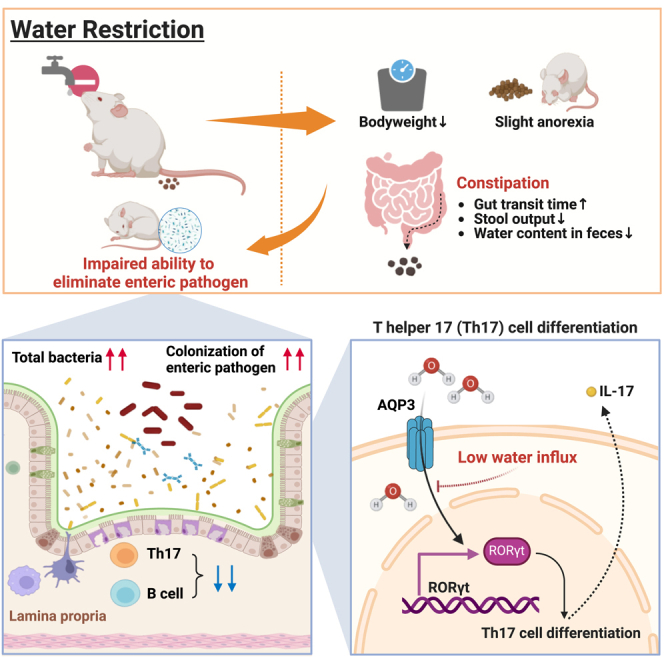

## Introduction

Water is the largest component of the human body, comprising more than 50% of body mass in a healthy adult and serving various functions in digestion, including absorption, transport of nutrients or waste, and thermoregulation.^1^ The three major sources for water acquisition include drinking, eating, and metabolism, with 70–80% water provided via beverages.^2^ Although the criteria for adequate water intake have been established in the United States,^3^ up to half of the adults are chronically underhydrated.^4^ Chronic underhydration affects human health and is associated with diseases, particularly metabolic disorders such as obesity, insulin resistance, and diabetes in adults.^5^ In addition, mice under chronic water restriction have been shown to exhibit a shortened lifespan, increased energy expenditure, cardiac fibrosis, and increased serum sodium levels.^6^ An elevated serum sodium concentration is associated with a risk of chronic diseases, including atherosclerosis, cardiovascular disease, and premature mortality.^6^^,^^7^^,^^8^^,^^9^

Water intake is also associated with gastrointestinal function. Low water intake reduces the fecal water content and is associated with an increased prevalence of functional constipation.^10^^,^^11^ Furthermore, differences in gut microbiota composition and metabolites have been observed between constipated patients and healthy individuals.^12^^,^^13^^,^^14^^,^^15^^,^^16^^,^^17^ Low water intake may therefore lead to direct or indirect changes in the gut microbial community. The relative abundance of several bacterial genera has been shown to differ between individuals with low and high water intake.^18^^,^^19^ These studies considered factors that influence gut microbiota composition, such as fluid intake, alcohol consumption, differences in dietary patterns, and exercise. Additionally, constipation is associated with an altered systemic immune response. Patients with constipation have been reported to exhibit an increased abundance of regulatory T (T_reg_) cells and spontaneous lymphocyte proliferation.^15^ In rats fasted overnight, the number of group 3 innate lymphoid cells (ILC3s) in the lamina propria of the jejunum decreased while that in the mesenteric lymph nodes increased 2 h after water intake.^20^ Thus, it is evident that water intake influences gastrointestinal function, gut microbiota, and immunity. However, the specific effects of water intake on the gastrointestinal tract remain largely unexplored. Therefore, in the current study, we aimed to investigate whether and how underhydration influences the gut microbiota and mucosal immunity in water-restricted mice.

## Results

### Water restriction induces constipation without dehydration

**Figure 1:**
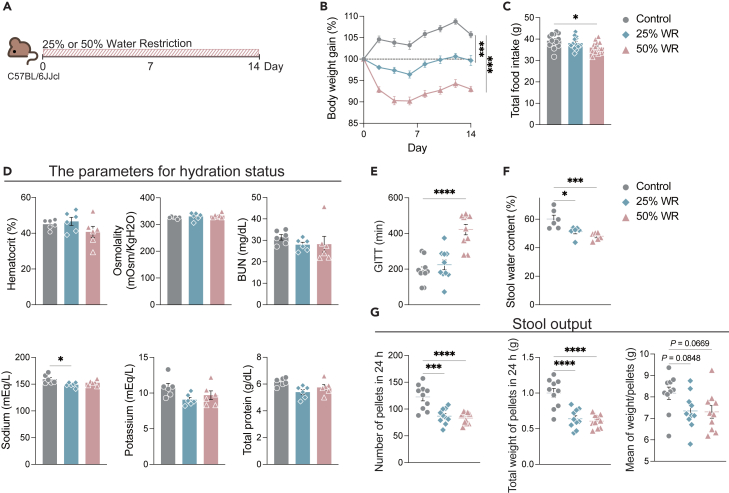
Water restriction suppresses body weight gain and induces constipation without dehydration (A) Diagram illustrating the protocol for water restriction. (B) Body weight gain compared with day 0. (*n* = 26 sample per group) (C) Total food intake during the regimen (*n* = 13 sample per group). (D–F) Hydration status, hematocrit, osmolality, blood urea nitrogen (BUN), sodium, potassium, and total protein in serum. (*n* = 6 sample per group) (E) Gastrointestinal transit time (GITT) was measured on day 13. (*n* = 10 sample per group) (F) Stool water content was measured on day 14. (*n* = 6 sample per group). (G) Stool output in 24 h on day 13. Left; number of pellets in 24 h, middle; weight of total pellets in 24 h, right; mean weight per pellet (*n* = 10 sample per group). Data pooled from independent tree experiments (B and C), and plots represent the mean and values ±S.E.M. One-way (C–G) or two-way ANOVA (B) followed by Dunnett’s test. ∗∗∗∗*p* < 0.0001, ∗∗∗*p* < 0.001, ∗∗*p* < 0.01, ∗*p* < 0.05.

To explore the impact of daily water intake on the gut, mice were subjected to chronic water restriction, as per previous research.^21^ Daily water intake during the acclimatization period was measured to determine normal water intake. Mice were then subjected to water restriction of 25% or 50% of the normal intake every day for 14 days (Figure 1A). The 25% and 50% water restrictions significantly decreased body weight gain, and the 50% water-restricted mice showed slight but significant anorexia (Figures 1B and 1C). These results were consistent with those obtained in previous research.^21^^,^^22^ However, the parameters for hydration status in the serum did not fluctuate due to water restriction (Figure 1D). As water intake can affect gut motility, stool consistency, and frequency, the gastrointestinal transit time (GITT), stool water content, and stool output were measured over 24 h. Fifty percent water restriction doubled the mean GITT (Figure 1E). The stool water content of water-restricted mice significantly decreased (Figure 1F). Additionally, the number and total weight of pellets at 24 h were significantly decreased in the 25% and 50% water-restricted mice (Figure 1G). Consistent with previous research,^10^^,^^11^ these results suggest that inadequate water intake induces constipation without dehydration.

### Water restriction induces an increase in the gut microbiota, altering its composition

**Figure 2:**
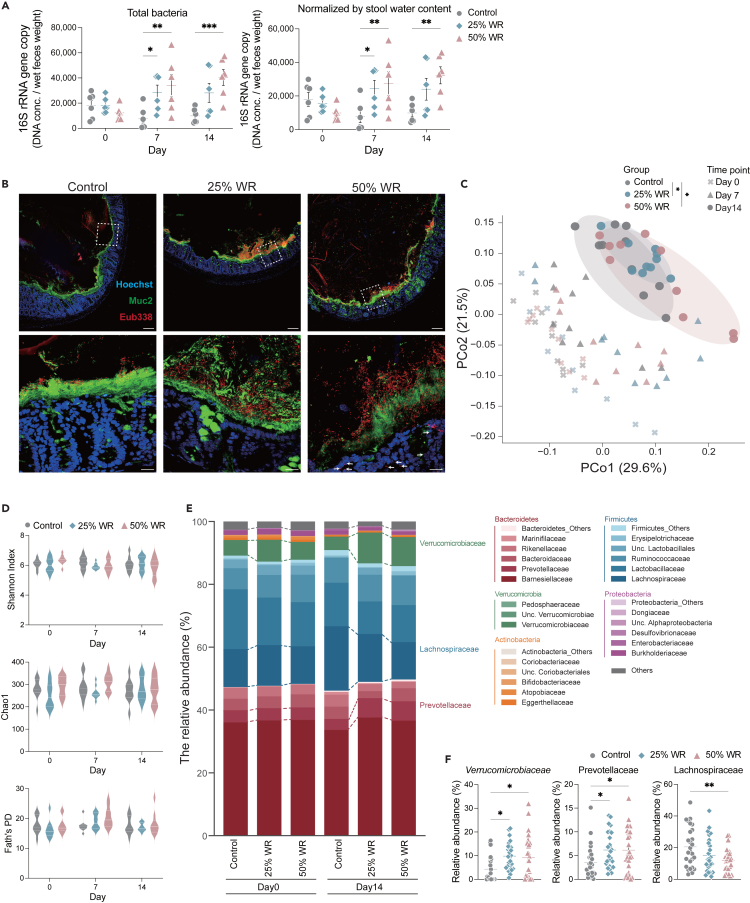
Water restriction increases the abundance and changes the composition of the gut microbiota (A) Left; Total bacteria in feces normalized to stool water content. Right; Total bacteria per mg of feces assessed via quantitative real-time PCR (qPCR) of the 16S rRNA gene (*n* = 6 sample per group). (B) A representative murine colon visualized via fluorescence *in situ* hybridization staining. Red; Bacteria (Eub338), Green; Muc2, Blue; Hoechst. Zoomed-in view of the region marked with a white dashed box. Scale bar; 100 μm. Scale bar of the zoomed-in panel, 20 μm. White arrows indicate bacterial intrusion into the colonic epithelial tissue (*n* = 6 sample per group). (C) PCoA (Principal Coordinate Analysis) plot based on UniFrac distance shows differences in the microbial community. Each symbol and color indicate the timepoint and group, respectively. (*n* = 10 sample per group) (D) The violin plot shows α-diversity on days 0, 7, and 14. Upper; Shannon index, middle; Chao1, lower; Faith’s phylogenetic diversity (*n* = 10 sample per group). (E) The stacked bar plot shows the microbiota composition on days 0 and 14. Each gradation color indicates the representative bacterial phylum families, respectively. Annotated bacterial families indicate significant differences in several major phyla compared with control on day 14 (*n* = 26 sample per group). (F) Bacterial families that exhibit significant differences and mean relative abundance in controls ≥1% in several major phyla (*n* = 26 sample per group). Data are pooled from independent tree experiments (D, E, and F), and plots represent the mean ± S.E.M. One-way ANOVA followed by Dunnett’s test. ∗∗*p* < 0.01, ∗*p* < 0.05 (A, E, and F). Pairwise PERMANOVA, ∗q-value <0.05 (C).

Next, we assessed whether water restriction can alter the gut microbiome structure. Water restriction increased the total bacterial count in feces (Figures 2A and 2B). This increase was not conferred by reduced stool water content (Figure 2A). Consistently, more dense bacteria and blurred mucus layers were noted in the 25% and 50% water-restricted mice than in control mice, as observed via fluorescence *in situ* hybridization (Figure 2B). Additionally, bacterial intrusion into the colonic epithelial tissue was observed in 50% water-restricted mice (Figure 2B). Furthermore, gut microbial communities were significantly altered in 25% and 50% of water-restricted mice compared to those in control mice, based on β-diversity (Figure 2C). While a prolonged gut transit time has been shown to alter the α-diversity of the gut microbiota,^23^^,^^24^^,^^25^ we observed no changes in α-diversity throughout the regimen (Figure 2D). Consistent with the results of α-diversity, the relative abundance of the major bacterial phylum was stable (Figure 2E). On the other hand, water restriction altered the abundance of specific bacterial families; the abundance of Verrucomicrobiaceae and Prevotellaceae increased while that of Lachnospiraceae decreased (Figures 2E and 2F). These results indicate that water restriction changes the gut microbial communities and density, thereby altering the gut mucosal structure.

### Water restriction decreases immune cell abundance, altering T cell function in the gut

**Figure 3:**
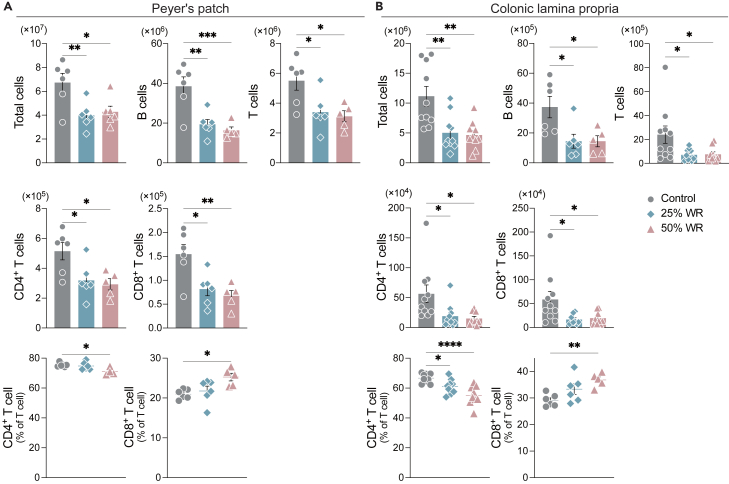
Water restriction decreases the number of immune cells and alters T cell function in the gut (A) Number and percentage of B cells (CD45^+^ B220^+^) and T cells (CD45^+^ CD3ε^+^), including CD4^+^ (CD45^+^ CD3ε^+^ CD4^+^) and CD8^+^ T cells, in PPs (*n* = 6 sample per group). (B) Number and percentage of B and T cells, including CD4^+^ T, and CD8^+^ T cells, in cLP (*n* = 6 sample per group). One-way ANOVA followed by Dunnett’s test. ∗∗*p* < 0.01, ∗*p* < 0.05.

Next, we assessed whether water restriction influences immune cell abundance and function in the Peyer’s patches (PPs) and colonic lamina propria (cLP). We found that water restriction decreased the abundance of immune cells, B cells, and T cells in both PPs and cLP, in parallel to the increased abundance of the gut microbiota (Figures 3A and 3B). Consistently, both CD4^+^ and CD8^+^ T cell counts decreased in water-restricted mice compared with those in control mice. However, the percentage of CD4^+^ T cells decreased, whereas that of CD8^+^ T cells increased (Figures 3A and 3B). The levels of total IgA in feces and serum were comparable between the control and water-restricted mice (Figure S1). These results suggest that underhydration impairs the maintenance of immune cells and alters their function in the gut.

### Water restriction diminishes the ability to eliminate *Citrobacter rodentium*

**Figure 4:**
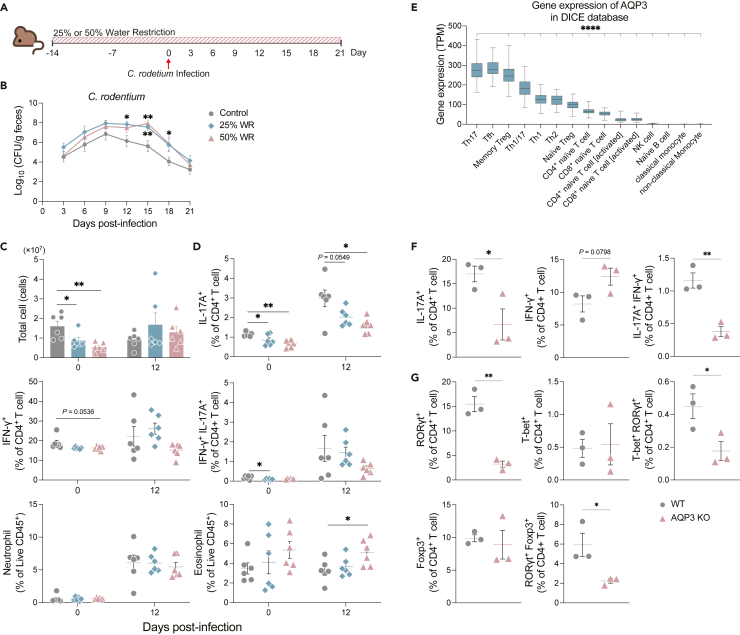
Water restriction diminishes the ability to eliminate the enteric pathogen *Citrobacter rodentium* (A) Diagram illustrating the protocol for water restriction and *C. rodentium* infection. (B) Pathogen colonization [colony-forming units (CFU/g feces)] in feces was assessed every 3 days post-infection. (*n* = 20 sample per group) (C) Total cells in the colonic lamina propria on days 0 and 12 post-infection (*n* = 6 sample per group). (D) T_h_17 (IL-17A^+^ CD4^+^ T cell), T_h_1 (IFN-γ^+^ CD4^+^ T cell), and IFN-γ^+^ T_h_17 (IL-17A^+^ IFN-γ^+^ CD4^+^ T cell) subpopulations among CD4^+^ T cells, neutrophils among the CD45^+^ population, and Ly6C^+^ macrophages among the CD45^+^ population (*n* = 6 sample per group). (E) AQP-3 gene expression in each immune cell subset in DICE (*n* = 80∼89 sample per immune cell subsets). (F) T_h_17 and T_h_1, IFN-γ^+^ T_h_17 subpopulations among CD4^+^ T cells in cLP (*n* = 3 sample per group). (G) RORγt^+^, T-bet^+^, T-bet^+^ RORγt^+^, Foxp3^+^, and RORγt^+^ Foxp3^+^ subpopulations among CD4^+^ T cells in the cLP of AQP-3-deficient mice (*n* = 3 sample per group). Plots represent the mean ± S.E.M. Unpaired t-test (F-G), one-way followed by Dunnett’s test (C–E), or two-way ANOVA (B) followed by Dunnett’s test. ∗∗∗∗*p* < 0.0001, ∗∗∗*p* < 0.001, ∗∗*p* < 0.01, ∗*p* < 0.05.

Since water restriction decreases the abundance of total immune cells and the T cell population, we hypothesized that these effects translate into an impaired enteric pathogen clearance. To test this hypothesis, we infected mice with *Citrobacter rodentium*, a mouse enteric pathogen cleared by CD4^+^ T and B cells^26^ (Figure 4A). *C. rodentium* was detected in the feces of inoculated mice at 3 days post-infection, with the *C. rodentium* burden further increasing and remaining elevated at days 6 and 9 post-infection, respectively. In control mice, the pathogen load started to decrease at 12 days post-infection and continued to decrease until day 21. In contrast, *C. rodentium* remained prominent until 15 days post-infection, while the fecal pathogen load eventually decreased from 18 days and further at 21 days after infection in water-restricted mice (Figure 4B). We also evaluated the immune responses against *C. rodentium* on days 0 and 12 post-infection. Before infection, the total cell number and percentage of IL-17A^+^ CD4^+^ T cells (T_h_17) and IFN-γ^+^ IL-17A^+^ T cells (IFN-γ^+^ T_h_17) in cLP were significantly lower in water-restricted mice than in control mice. After *C. rodentium* infection, the number of total cells and percentage of IFN-γ^+^ T_h_17 cells in the cLP of the water-restricted mice were comparable with those in control mice. However, the percentage of T_h_17 cells and IFN-γ^+^ T_h_17 cells remained lower in the water-restricted mice than in control mice. Relative to controls, the percentage of IFN-γ^+^ CD4^+^ T cells (T_h_1) tended to decrease in water-restricted mice before infection and was comparable in all groups on day 12 post-infection. The percentage of innate immune cells, including neutrophils and Ly6C^+^ macrophages, increased after infection but was not different in all groups before and after infection (Figures 4C and 4D). IL-22-producing CD4^+^ T cells and ILC3 also play a crucial role in host defenses in the early phases of infection.^27^^,^^28^ However, consistent with the previous reports and our results of the comparable burden of *Citrobacter rodentium* in the early infection phase, the proportions of IL-22^+^ CD4^+^ T cells and IL-22^+^ ILC3 were maintained at an equal level in cLPs of water-restricted mice (Figure S2). Additionally, the serum levels of *C. rodentium*-specific IgG did not differ between water-restricted and control mice (Figure S3). Taken together, these results indicate that underhydration impairs the ability to eliminate the enteric pathogen *C. rodentium* due to an attenuated T_h_17 cell response. To elucidate whether gut dysbiosis induced by water restriction leads to impaired development and maintenance of T_h_17 cells in the colon, fecal microbiota transplantation (FMT) was performed in antibiotics-treated mice. The water-restricted donor mice exhibited fewer CD4^+^ T cells including T_h_17 cells in cLPs than the control donor mice. However, FMT from the water-restricted donor mice into antibiotics-treated mice did not impair T_h_17 cells but increased CD4^+^ T cells compared to those from control donor mice (Figure S4). Thus, changes in gut microbiota composition induced by water restriction did not impair the development or maintenance of T_h_17 cells in cLPs.

### Water channel protein aquaporin 3 is required for the maintenance of T_h_17 in the gut

To reveal the mechanisms of how water restriction impairs the T_h_17 in cLPs, we focused on the direct relations between water influx and immune cells. It has been shown that T cells switch from metabolic water gain to water influx during the slow-growth to fast-growth phase^29^ and that CD4^+^ T cells, especially follicular CD4^+^ T cells (T_fh_), require water influx via aquaporin 3 (AQP-3) for their proliferation and T cell-dependent antibody response.^30^ Additionally, T_h_17 cells highly express AQP-3 during their differentiation.^31^ Therefore, we investigated whether adequate water intake maintains intestinal T_h_17 cells in an AQP-3-dependent manner. Consistent with previous reports, human T_h_17 and T_fh_ cells showed significantly higher AQP-3 expression than other immune cell subsets as per DICE (Database of Immune Cell Expression, Expression quantitative trait loci (eQTLs), and Epigenomics)^32^ (Figure 4E). To clarify the role of AQP-3 in the gut T_h_17 cell population, we compared CD4^+^ T cell subsets in the cLP from wild-type and AQP-3-deficient mice. Notably, the percentage of T_h_17 and IFN-γ^+^ T_h_17 cells in the cLP was significantly lower in AQP-3-deficient mice than in wild-type mice, but there was no significant difference in T_h_1 cells (Figure 4F). The function and differentiation of T_h_17 cells are regulated by the nuclear hormone receptor retinoic-acid-receptor-related orphan nuclear receptor-γt (RORγt), a transcription factor.^33^^,^^34^ Thus, we examined RORγt expression in CD4^+^ T cell subsets. In addition to the percentage of RORγt^+^ CD4^+^ T cells, that of RORγt^+^ T-bet^+^ CD4^+^ T cell and RORγt^+^ Foxp3^+^ CD4^+^ T cells was significantly decreased in AQP-3-deficient mice compared to that in control mice. There were no differences in the percentages of T-bet^+^ CD4^+^ T cells or Foxp3^+^ CD4^+^ T cells between the groups (Figure 4G). These results indicate that AQP-3 is required for the functional maintenance and differentiation of RORγ^+^ T cells, including T_h_17, in the gut.

## Discussion

In this study, we revealed the adverse effects of chronic water restriction on gastrointestinal homeostasis, characterized by an increase in the number and changes in the composition of gut microbiota, compromised T_h_17 cell function and differentiation, as well as limited capacity for the elimination of enteric pathogens.

Consistent with previous research,^21^^,^^22^ water restriction suppressed body weight gain, and 50% of water-restricted mice showed significant anorexia. On the other hand, it has been reported that mice subjected to 30% water restriction exhibit higher food consumption and energy expenditure two months after implementation.^6^ Thus, the anorexia induced by water restriction may be temporary, with chronic rehydration giving rise to different feeding behaviors.

Water restriction-induced constipation, which was accompanied by a lower water content in feces and prolonged GITT. Together with previous findings,^10^^,^^11^ these results indicate that limited water intake can induce constipation. GITT and stool consistency are associated with α-diversity and enterotype.^25^^,^^35^^,^^36^^,^^37^ We found that the total bacterial counts in feces were significantly increased in water-restricted mice compared to those in control mice. Additionally, the gut microbial communities differed between control and water-restricted mice, without significant differences in α-diversity and the relative abundance of major bacterial phylum. The relative abundance of Prevotellaceae increased in water-restricted mice, but the abundance *Prevotella*, which belongs to the Prevotellaceae family, is not associated with colonic transit time.^24^^,^^25^ On the other hand, *Prevotella* species in the mucosal site are related to various inflammations including colitis.^38^^,^^39^ Thus, it is possible that the increased relative abundance of Prevotellaceae in our study was caused by other factors, such as changes in gut mucosal integrity, rather than by GITT and stool consistency. In contrast, water-restricted mice showed an increased relative abundance of Verrucomicrobiaceae. Consistent with this result, the abundance of *Akkermansia*, which belongs to the Verrucomicrobiaceae family, is positively correlated with GITT,^35^^,^^36^ and these bacteria are enriched in healthy individuals with poor hydration status.^19^ *Akkermansia* is a mucin-degrading bacterium^40^^,^^41^ residing within the mucus layer.^42^^,^^43^ The thinner mucus layer induced by mucin-degrading bacteria, including *Akkermansia*, impairs the intestinal barrier, rendering it penetrable by bacteria^44^^,^^45^ and aggravating colitis.^46^^,^^47^ The blurred colonic mucus layer in water-restricted mice is probably associated with a greater abundance of the Verrucomicrobiaceae family members, including *Akkermansia*. Lachnospiraceae comprises a variety of characteristic bacterial genera,^48^ such as *Clostridium*, *Blautia*, and *Lachnospira*,^49^ and these bacteria genera are associated with both beneficial and harmful effects on health.^49^ Although changes in the gut microbiota composition by water restriction did not directly influence the decrease in T_h_17, whether the increase in the total bacteria number and bacterial intrusion into colonic tissues could affect gut mucosal immunity should be clarified.

The total cell counts as well as counts of CD4^+^/CD8^+^ T cells and B cells in PPs and cLP were decreased, with *C. rodentium* clearance delayed in water-restricted mice compared to that in control mice. We also observed that the percentage of T_h_17 cells was lower before and after infection in mice subjected to water restriction. IL-22-producing CD4^+^ T cells and ILC3 also play an important role in early host defense against *C. rodentium*.^27^^,^^28^^,^^50^ However, the percentage of IL-22^+^ CD4^+^ T cells and IL-22^+^ ILC3 was maintained at an equal level in the water-restricted mice compared to the control group on days 12 post-infection. The results of decreased IL-17A^+^ CD4^+^ T cells but not IL-22^+^ CD4^+^ T cells and IL-22^+^ ILC3, as well as impaired peak phase clearance of *C. rodentium* in the water-restricted mice, are consistent with that IL-17A^+^ subpopulations increase in peak phase^28^^,^^51^ and IL-17A and IL-17R signaling are implicated in the clearance of *C. rodentium*.^52^^,^^53^ Thus, impaired pathogen elimination in water-restricted mice may be due to a reduced number of T_h_17 cells in the gut.

As supporting our hypothesis, the percentage of T_h_17 (RORγt^+^ CD4^+^) cells as well as RORγt^+^ T-bet^+^ CD4^+^ T cells and RORγt^+^ FoxP3^+^ CD4^+^ T cells was significantly decreased in the cLP of AQP-3-deficient mice compared to that in WT mice. CD4^+^ T cell subsets require water influx via AQP-3 for proliferation or differentiation.^29^^,^^30^^,^^31^^,^^54^ In addition, the DICE database indicated that T_h_17 cells express higher levels of AQP-3 than other immune cell subsets. These observations suggest that water influx and AQP-3 are required for RORγt expression and the maintenance of T_h_17 cells, thereby enhancing the capacity for enteric pathogen elimination. To assess whether AQP3 is required for the development and maintenance of T_h_17 cells under adequate water intake, it is imperative to verify the impacts of AQP3 deficiency on the decrease in Th17 cells by water restriction. Regardless, considering that the expressing RORγt^+^ cells including T_h_17, ILC3, NKT17, γδ-T, and mucosal-associated invariant T cells explicitly express AQP3,^55^^,^^56^^,^^57^ our results emphasize the importance of water influx into RORγt expressing cells for their development and maintenance via AQP3.

Taken together, the current findings indicate that water restriction impairs the elimination of enteric pathogens owing to a prolonged gut transit time and fewer T_h_17 cells within the gut. In addition, we reveal a mechanism in the regulation of IL-17A^+^ T cells and RORγt expression. Our findings shed light on the significance of optimal water intake in maintaining intestinal homeostasis and gut immunity.

### Limitations of the study

Water restriction decreased the number and impaired the function of Th17 cells. The difficulties in confirming whether water influx via AQP-3 is reduced under water restriction and how this influx affects RORγt expression represent one of the limitations. To clarify this, other approaches such as regulating water influx into the intracellular space are required. Elucidating the relationship between water abundance and the RORγt protein may shed light on more specific mechanisms.

Finally, reports linking inadequate water intake to pathogen elimination and infection in humans are scarce. Thus, investigating the relationship between daily water intake and infection to validate our findings in humans is crucial for understanding their potential impact on health.

## STAR★Methods

### Key resources table

**Table:** 

REAGENT or RESOURCE	SOURCE	IDENTIFIER
**Antibodies**		

Purified anti-mouse CD16/32 Antibody (93)	BioLegend	Cat# 101302, RRID: AB_312801
BD Horizon™ V450 Hamster anti-Mouse CD3e (500A2)	BD Biosciences	Cat# 560804, RRID: AB_2034005
Brilliant Violet 421™ anti-mouse/human CD45R/B220 Antibody	BioLegend	Cat# 103251, RRID: AB_2562905
Brilliant Violet 510™ anti-mouse CD45 Antibody (30-F11)	BioLegend	Cat# 103138, RRID: AB_2563061
PE, ROR gamma (t) Monoclonal Antibody (B2D)	Thermo Fisher Scientific	Cat# 12-6981-82, RRID: AB_10807092
PerCP-Cyanine5.5, FOXP3 Monoclonal Antibody (FJK-16s)	Thermo Fisher Scientific	Cat# 45-5773-82, RRID: AB_914351
PE-Cyanine7, CD4 Monoclonal Antibody (GK1.5)	Thermo Fisher Scientific	Cat# 25-0041-82, RRID: AB_469576
eFluor™ 660, CD8a Monoclonal Antibody (53-6.7)	Thermo Fisher Scientific	Cat# 50-0081-80, RRID: AB_10597596
APC, anti-mouse CD8a Antibody (53-6.7)	BioLegend	Cat# 100712, RRID: AB_312751
APC, anti-mouse CD3ε Antibody (145-2C11)	BioLegend	Cat# 100312, RRID: AB_312677
APC, ROR gamma (t) Monoclonal Antibody (B2D),	Thermo Fisher Scientific	Cat# 17-6981-82, RRID: AB_2573254
FITC, anti-mouse CD4 Antibody (RM4-5)	BioLegend	Cat# 100510, RRID: AB_312712
PE-Cyanine7, IL-17A Monoclonal Antibody (eBio17B7)	Thermo Fisher Scientific	Cat# 25-7177-82, RRID: AB_10732356
PE, IFN gamma Monoclonal Antibody (XMG1.2)	Thermo Fisher Scientific	Cat# 12-7311-82, RRID: AB_466193
PerCP-Cyanine5.5, T-bet Monoclonal Antibody (eBio4B10 (4B10))	Thermo Fisher Scientific	Cat# 45-5825-82, RRID: AB_953657
Fixable Viability Stain 780	BD Biosciences	Cat# 565388, RRID: AB_2869573
Hoechst 33342	Thermo Fisher Scientific	Cat# H1399
MUC2 Antibody	Novus Biologicals	Cat# NBP1-31231, RRID: AB_10003763
Alexa Fluor® 488 AffiniPure Donkey Anti-Rabbit IgG (H + L)	Jackson ImmunoResearch	Cat# 711-545-152, RRID: AB_2313584
BV510 Rat Anti-Mouse Ly-6G (1A8)	BD Biosciences	Cat# 740157, RRID: AB_2739910
Alexa Fluor® 488 anti-mouse CX3CR1 Antibody (SA011F11)	BioLegend	Cat# 149022, RRID: AB_2565705
PE, CD11b Monoclonal Antibody (M1/70)	Thermo Fisher Scientific	Cat# 12-0112-83, RRID: AB_273487005
PE-CF594, Rat Anti-Mouse Siglec-F (E50-2440)	BD Biosciences	Cat# 562757, RRID: AB_2687994
PE/Cyanine7, anti-mouse F4/80 Antibody (BM8)	BioLegend	Cat# 123114, RRID: AB_893478
APC, Rat Anti-Mouse Ly-6C (AL-21)	BD Biosciences	Cat# 560595, RRID: AB_1727554
Rabbit Anti-Mouse IgG H&L	Abcam	Cat# ab97046, RRID: AB_956006

**Bacterial and virus strains**		

*Citrobacter rodentium* stain DBS120 (pCRP1: Tn5)	Kim et al.^58^	N/A

**Chemicals, peptides, and recombinant proteins**		

Cell Activation Cocktail (without Brefeldin A)	BioLegend	Cat# 423302
Protein Transport Inhibitor Cocktail (500X)	Thermo Fisher Scientific	Cat# 00-4980-03
Liberase™ Research Grade	Roche Diagnostics	Cat# 26628-22-8
Newborn calf serum	Gibco	Cat# 16010-159
Deoxyribonuclease I from bovine pancreas	Sigma-Aldrich	Cat# DN25-5G
Albumin, Bovine Serum, Globulin-free	Nacalai tesque	Cat# 01281-26
Dithiothreitol	Nacalai tesque	Cat# 14128-62
MacConkey Agar Base	Difco™	Cat# 281810
Kanamycin Monosulfate	Nacalai tesque	Cat# 08976-84
TB Green premix Ex Taq (Tli RNaseH Plus)	TaKaRa	Cat# RR420A
Qubit™ dsDNA Quantification Assay Kits	Thermo Fisher	Cat# Q32854
Donkey serum	Sigma-Aldrich	Cat# D9663-10ML
10% SDS Solution	Nippon gene	Cat# 311-90271
ProLong™ Gold Antifade Mountant	Thermo Fisher	Cat# P36930
cOmplete™, Mini Protease Inhibitor Cocktail	Roche	Cat# 11836153001
LB Broth, Lennox	Nacalai tesque	Cat# 20066-95

**Critical commercial assays**		

MagDEA®Dx SV	Precision System Science	Cat# E1300
Mouse IgA ELISA Kit	Bethyl Laboratories	Cat# E90-103
Transcription Factor Buffer Set	BD Pharmingen™	Cat# 562574
SureBlue™ TMB 1-Component Microwell Peroxidase Substrate	LGC Clinical Diagnostics, Inc (SeraCare)	Cat# 5120-0077
TMB Stop Solution	LGC Clinical Diagnostics, Inc (SeraCare)	Cat# 5150-0021

**Deposited data**		

Mouse: C57BL/6J	CLEA Japan Inc.	N/A
Aquaprin-3 deficient mice	Ma et al.^59^	N/A

**Oligonucleotides**		

16S universal primer Fwd: CCA AAC TCC TAC GGG AGG CAG CAG	This study	N/A
16S universal primer Rvs: CAT GGA CTA CCA GGG TAT CTA ATC	This study	N/A
Total bacteria primer Fwd: TCC TAC GGG AGG CAG CAG	This study	N/A
16S universal primer Rvs: GGA CTA CCA GGG TAT CTA ATC CTG TT	This study	N/A
TAMURA-labelled Eub338 probe: GCT GCC TCC CGT AGG AGT	This study	N/A

**Software and algorithms**		

GraphPad Prism version 10.0.3	GraphPad Software	https://www.graphpad.com/
QIIME2 Version 2022.2.0	QIIME	https://qiime2.org/
Python Version 3.8.3	Python	https://www.python.org/
Matplotlib Version 3.5.0	matplotlib	https://matplotlib.org/
Seaborn Version 0.11.2	seaborn	https://seaborn.pydata.org/#
Flowjo Version 10	FlowJo LLC	https://www.flowjo.com/
Database of Immune Cell Expression, Expression quantitative trait loci (eQTLs), and Epigenomics	Schmiedel et al.^32^	Accession: phs001703.v1.p1 https://dice-database.org/

**Other**		

Mouse diet: CE-2	CLEA Japan Inc.	N/A
SpectraMax iD3	Molecular Devices	https://www.moleculardevices.co.jp/systems/spectramax-id3-multi-mode-microplate-reader#gref
magLEAD 12gc	Precision system science	https://www.pss.co.jp/product/magtration/lead6-12gc.html
MACSQuant	Miltenyi Biotec	https://www.miltenyibiotec.com/JP-en/products/macs-flow-cytometry/flow-cytometers.html#gref
DLAB DM1424 Hematocrit Centrifuge	DLAB	https://www.dlabsci.com/productDetail?id=61bcdb77ac7dea621e5026d890c90863
Freeze dryer VD-550R	TAITEC	Cat# 0067047-000
StepOnePlus	Thermo Fisher Scientific	https://www.thermofisher.com/order/catalog/product/4376598?SID=srch-srp-4376598
Qubit 2.0 Fluorometer	Thermo Fisher Scientific	https://www.thermofisher.com/jp/ja/home/brands/product-brand/qubit/qubit-fluorometer.html
gentleMACS™ Octo Dissociator with Heaters	Miltenyi Biotec	Cat# 130-096-427
gentleMACS™ M Tubes	Miltenyi Biotec	Cat# 130-093-236

### Resource availability

#### Lead contact

Further information and requests for resources and reagents should be directed to and will be fulfilled by the lead contact, Yun-Gi Kim (kim.yungi@kitasato-u.ac.jp).

#### Materials availability

This study did not generate new unique reagents.

#### Data and code availability

• All data reported in this paper will be shared by the lead contact upon request.
• This paper does not report original code.
• Any additional information required to reanalyze the data reported in this paper is available from the lead contact upon request.
• Human gene expression data sets from DICE database are available to access at https://dice-database.org. The accession number for individual data sets is: phs001703.v1.p1.

### Experimental model and study participant details

#### Mice

Eight-to ten-week-old female and male C57BL/6JJcl mice (CLEA Japan Inc.) and AQP-3-deficient mice^59^ were housed under standard conditions, with controlled lights (12 h light, 12 h dark cycle), temperature (24 ± 0.5°C), and humidity (40 ± 5%), at the animal facilities of the Faculty of Pharmacy, Keio University (Tokyo, Japan). Mice had free access to food, CE-2 (CLEA Japan, Inc.), and filter-sterilized drinking water (tap water). Following a 5–7-day acclimatization period, the mice were randomly assigned to three groups (*Ad libitum*, 25% Water Restriction, 50% Water Restriction). The mice were rotated between cages to reduce variations in the gut microbiome composition caused by the housing environment. The similar phenotypes were observed in depending on murine sexes. All experiments were approved by the ethics committees of Keio University.

#### Water restriction

The conditions of water restriction were as in a previous study,^21^ in which chronic water restriction was performed without dehydration. Daily water intake was measured during the acclimatization period, and normal water intake was determined. Subsequently, water was supplied at 25% or 50% of normal water intake every day.

#### Citrobacter rodentium infection

The kanamycin-resistant WT *Citrobacter rodentium* strain DBS120 (pCRP1 Tn5)^58^ was grown overnight in 50 μg/mL Luria-Bertani (LB) broth with shaking at 37°C. Mice were infected with 0.2 mL of phosphate-buffered saline (PBS) containing approximately 1×10^9^ colony-forming units (CFU) of C. *rodentium* via oral administration. To determine the bacterial load in feces, murine fecal pellets were collected every 3 days, homogenized in PBS, and plated after serial dilutions on McConkey agar containing 50 μg/mL kanamycin; CFUs were determined after a 48-h incubation at 37°C.

#### The comparison of Human gene expressions of aquaporin 3 from DICE database

The public datasets of AQP3 gene expression in human immune cells were provided and compared at DICE (Database of Immune Cell Expression, Expression quantitative trait loci (eQTLs), and Epigenomics)^32^ (https://dice-database.org). The accession number for individual datasets is: phs001703.v1.p1.

### Method details

#### Hydration status parameters

Blood samples were obtained through cardiac puncture under anesthesia, collected in capillary tubes coated with heparin, and separated via centrifugation at 12000×g and 28°C for 5 min using a hematocrit centrifuge (DLAB). Hct was determined using the ratio of the precipitate. Blood osmolality and serum concentrations of sodium, potassium, total protein, and blood urea nitrogen (BUN) were measured by Oriental Yeast Co., Ltd.

#### Stool output, GITT, and fecal water contents

To measure the stool output and GITT, the mice were kept in individual cages without bedding chips with a stainless-steel mesh floor to avoid coprophagia. Stool output was quantified as the number of fecal pellets, and the mean weight was calculated for 24 h.

To measure GITT, Carmine red (Wako Pure Chemical Industries) was prepared as a 6% (w/v) solution of 0.5% methylcellulose. The mice were orally administered 300 μL Carmine red solution and monitored until the first red fecal pellet appeared.^60^

The weights of the fecal pellets were measured before and after overnight lyophilization and calculated according to the following equation:

$$Fecal\;water\;contents\;\left(\%\right)=\frac{wet\;weight-dried\;weight}{wet\;weight}\times 100$$

#### 16S rRNA sequencing and analysis

Bacterial DNA was extracted from mouse feces using the E.Z.N.A. Stool DNA Kit Pathogen Detection Protocol (OMEGA) and purified using a magLEAD 12gc nucleic acid extraction instrument (Precision System Science Co., Ltd.). DNA was amplified via PCR using primers specific to the V3-V4 regions of the 16S rRNA gene (key resources table). Sequencing was performed by Cancer Precision Medicine (Japan) on a MiSeq System (Illumina Inc.). Raw FASTQ files were processed using QIIME2 (Version 2022.2.0)^61^ with denoising via DADA2.^62^ Taxonomy was assigned using the DADA2 implementation of the RDP classifier in SILVA.^63^^,^^64^

#### Total bacterial count

To measure the total bacterial count in murine feces, quantitative real-time PCR (qPCR) was performed using 16S rRNA primer pairs^65^^,^^66^ (key resources table). qPCR amplification and detection were performed in Applied Biosystems (Thermo Fisher Scientific). Each reaction mixture (20 μL) contained 10 μL TB Green premix Ex Taq (Takara Inc.), 0.4 μL of each primer, 7.2 μL distilled water, and 2 μL extracted DNA. The qPCR conditions were 95°C for 15 s, 60°C for 1 min, and 95°C for 15 s, followed by 35 cycles of 95°C for 5 s, and 60°C for 30 s. Standard curves were obtained using serial 2-fold dilutions of DNA extracted from *Escherichia coli*, and DNA concentration was measured using a Qubit 2.0 Fluorometer (Thermo Fisher Scientific).

#### Isolation of cells from the cLP and PPs

Colon sections with the intestinal contents removed were washed twice in D-PBS and separated into intraepithelial cells and other components through incubation in HBSS (−) containing 1 mM dithiol-threitol and 20 mM EDTA. Colon sections were cut into tiny fragments, and cells were isolated via incubation in a digestion cocktail containing 0.125 mg/mL DNase I (Merck, Darmstadt, Germany) and 0.2 U/mL Liberase (Roche Diagnostics, Mannheim, Germany), followed by washing with RPMI1640 containing 2% newborn calf serum (NBCS) (Thermo Fisher Scientific, Waltham, MA, USA). Mononuclear cells were isolated via gradient centrifugation using Percoll (GE Healthcare, Chicago, Illinois, US).

PPs were cut from the intestine, and dissociation was performed in gentleMACS Octo Dissociate with Heaters (Miltenyi Biotec) and gentleMACS M tubes (Miltenyi Biotec), adding 2 mL RPMI1640 containing 2% NBCS. The dissociation setting was offered by Miltyeyi Inc. Dissociated PPs were collected via centrifugation at 500 ×g and 4°C for 1 min. After filtration through a 100 μm cell strainer, the cells were resuspended in D-PBS (−) containing 2% NBCS.

#### Flow cytometry analysis

To determine cytokine expression, isolated cells were stimulated with cell stimulation medium (containing cell activation cocktail (BioLegend) and protein transport inhibitor cocktail (eBioscience) in RPMI supplemented with 5% inactivated fetal bovine serum, 1 mM L-glutamine, 1 M HEPES, 55 mM 2-mercaptoethanol, 10,000 units/mL penicillin, and 100 mg/mL streptomycin) for 4 h. After stimulation, the cells were stained with various antibodies. For surface and intracellular staining, nonspecific binding was blocked with Fc anti-CD16/32 antibody (1:200) in D-PBS (−) containing 2% NBCS before staining with fluorochrome-conjugated antibodies (key resources table). The cells were fixed, permeabilized, and stained with antibodies for intracellular staining using the Transcription Factor Buffer Set (BD Biosciences) according to the manufacturer’s instructions. Fixable Viability Stain 780 (BD Biosciences) was used to discriminate dead cells. Stained samples were analyzed using a MACSQuant flow cytometer (Miltenyi Biotech) and FlowJo software (version 10, FlowJo LLC, Ashland, OR, USA).

#### Fluorescence *in situ* hybridization

Colon sections were fixed in methanol-Carnoy’s solution and embedded in paraffin. The colon slices were hybridized with a TAMURA-labeled Eub338 probe (key resources table) in a hybridization solution including 5 M NaCl, 1 M Tris-HCL, formamide, and nuclease-free water at 50°C overnight after deparaffinizing. After washing, the colon slices were blocked with 10% donkey serum (Sigma Aldrich) for 15 min and treated with a rabbit muc2 polyclonal antibody (1:100; Novus Biologicals) in the dark at room temperature overnight. After washing, colon slices were visualized using Alexa Fluor 488 anti-rabbit IgG (1:500; Jackson ImmunoResearch) and Hoechst 33342 (1:200; Thermo Fisher Scientific) for 3 h at room temperature in the dark. Colon slices were then enclosed in ProLong Gold Antifade Mountant (Invitrogen).

#### Total IgA ELISA

Fecal samples were lyophilized overnight. Freeze-dried feces (10 mg) were disrupted with 3.0 mm Zirconia Beads (Biomedical Science) and a Complete Mini Protease Inhibitor Cocktail (Roche, Basel, Switzerland) by vigorous shaking (1,500 rpm for 15 min) using a Shake Master (Biomedical Science), followed by centrifugation at 14000 rpm and 4°C for 15 min to collect the supernatant as a fecal extract. The samples were stored at −80°C. To measure total IgA levels, a Mouse IgA ELISA Quantitation Set (Bethyl Laboratories) was used.

#### Fecal microbiota transplantation

The recipient mice were treated with antibiotics^67^ (1 mg/mL ampicillin (Nacalai Tesque, Inc., Kyoto, Japan) and 0.5 g/mL vancomycin (FUJIFILM Wako Pure Chemical Corp., Osaka, Japan)) for 2 weeks (Figure S2A). The flesh fecal pellets collected from each donor mouse were merged and suspended at 10 mg/mL with D-PBS under anaerobic conditions. After precipitating the debris by incubation for 10 min, fecal supernatants were collected. Subsequently, recipient mice received 100 μL fecal supernatant.

#### *C. rodentium*-specific IgG ELISA

To coat wells with the *C. rodentium* antigen, the kanamycin-resistant WT *C. rodentium* strain DBS120 (pCRP1 Tn5)^58^ was grown overnight in 50 μg/mL LB broth with shaking at 37°C. *C. rodentium* resuspended in sonication buffer containing a Complete Mini Protease Inhibitor Cocktail (Roche, Basel, Switzerland) in PBS was sonicated for 5 min after being heat-killed for 1 h at 60°C.^68^^,^^69^^,^^70^^,^^71^ *C. rodentium* antigen was applied to an ELISA plate at 100 μL/well and incubated overnight. After washing with 0.5% tween 20 in PBS, the wells were blocked with 1% bovine serum albumin in PBS for 1 h at room temperature. After washing three times, 100 μL/well serially diluted serum samples were added and incubated for 2 h at room temperature. Horseradish peroxidase (HRP)-conjugated anti-mouse IgG (Abcam) was added to the well and incubated for 1 h. After washing the plate, bound antibodies were detected using 3,3′,5,5′-tetramethylbenzidine (LGC Clinical Diagnostics, Inc (SeraCare)). After 15 min, the reaction was stopped using TMB stop solution (SeraCare; LGC Clinical Diagnostics, Inc (SeraCare)). Absorbance was determined using a SpectraMax iD3 (MOLECULAR DEVICES) at 450 and 570 nm.

#### Statistical analysis

Dunnett’s test was used for statistical analyses of the two groups. Statistical analyses were performed using GraphPad Prism software (version 10.0.3, GraphPad Software Inc.). Differences with ∗: *p* < 0.05, ∗∗: *p* < 0.01 were considered statistically significant. Data were visualized using Python (version 3.8.3), matplotlib (version 3.5.0), and seaborn (version 0.11.2) packages as well as GraphPad Prism software.

## Supplemental information

Document S1. Figures S1‒S4
