# Supplementary material for: Sufficient water intake maintains the gut microbiota and immune homeostasis and promotes pathogen elimination
Source: iScience. 2024 May 3;27(6):109903. doi: 10.1016/j.isci.2024.109903 (PMC11126815; doi:10.1016/j.isci.2024.109903)
Supplement: Document S1. Figures S1‒S4 [file mmc1.pdf]

iScience, Volume *27*

Supplemental information

Sufficient water intake maintains the gut microbiota and immune homeostasis and promotes pathogen elimination

Kensuke Sato, Mariko Hara-Chikuma, Masato Yasui, Joe Inoue, and Yun-Gi Kim

Supplemental Information

This PDF file includes:

Figures S1 and S4

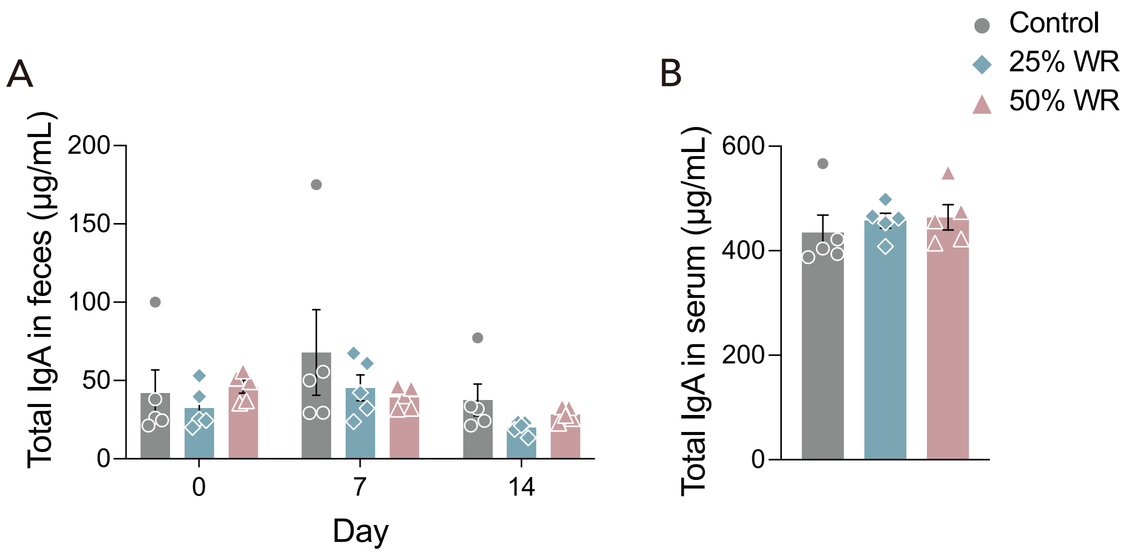

Figure S1. Total IgA levels are not affected by water restriction, related to Figure 3.

(A) Total IgA level in feces. (B) Total serum IgA level over 14 days. Plots represent the mean ± S.E.M. One-way ANOVA followed by Dunnett's test (B) or two-way ANOVA (A) followed by Dunnett's test.

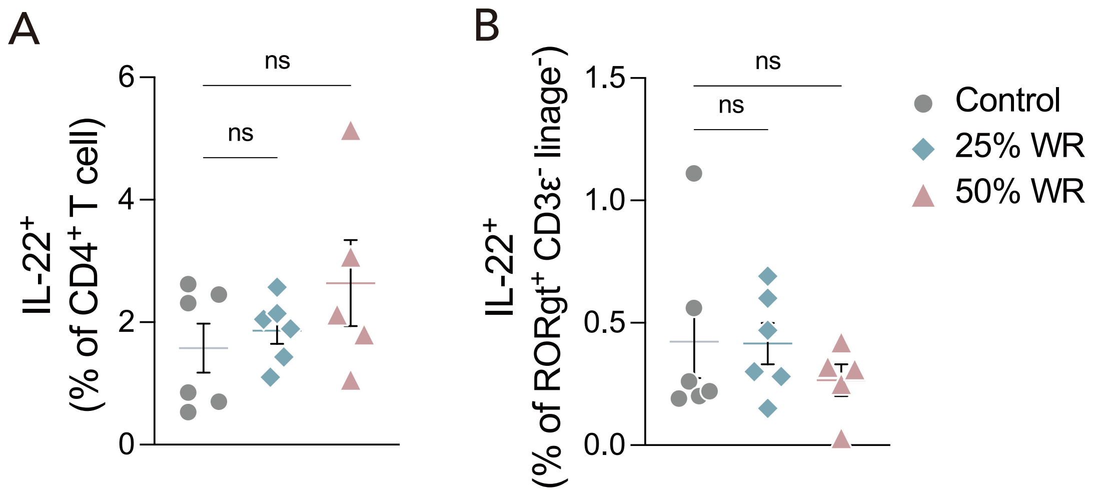

Figure S2. IL-22$^+$ CD4$^+$ T cells and ILC3 were maintained in water-restricted mice, related to Figure 4.

(A) IL-22$^+$ subpopulations among CD4$^+$ T cells in cLPs on days 12 post-infection. (B) IL-22$^+$ subpopulations among ILC3 (CD45$^+$ CD3ε$^-$ linage$^-$ RORγt$^+$) in cLPs on days 12 post-infection. Plots represent the mean and values ± S.E.M. One-way ANOVA followed by Dunnett's test (A and B).

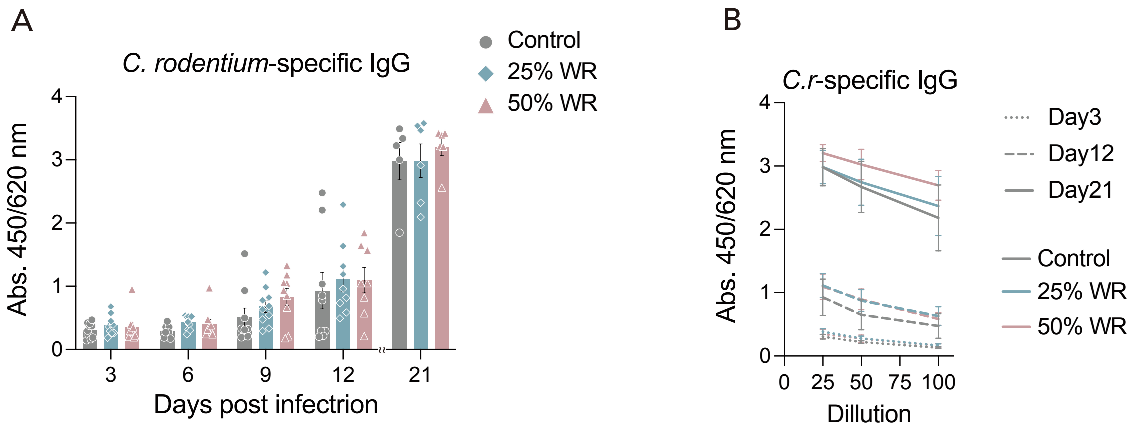

Figure S3. The *C. rodentium*-specific antibody response is maintained during water restriction, related to Figure 4.

(A) Temporal *C. rodentium*-specific IgG levels in *C. rodentium*-infected mice. (B) *C. rodentium*-specific IgG antibody titer in serum. Plots represent the mean and values ± S.E.M. One-way ANOVA followed by Dunnett's test (A) or two-way ANOVA (B) followed by Dunnett's test.

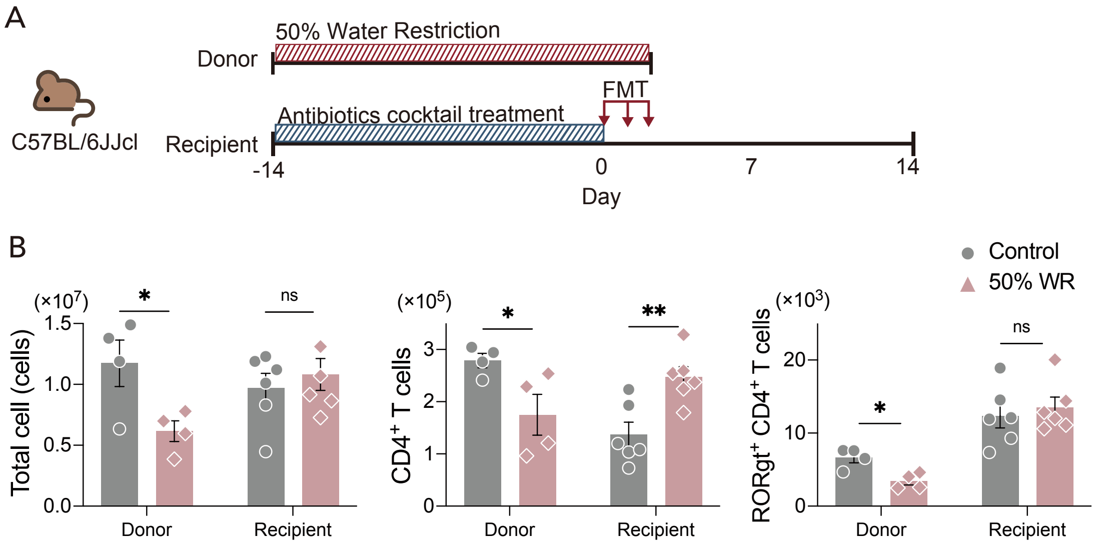

Figure S4. The microbiome altered by water restriction did not decrease Th17 cells in cLPs, related to Figure 3.

(A) Diagram illustrating the protocol for fecal microbiota transplantation (FMT). (B) The number of total cells, $CD4^+$ T cells, $ROR\gamma t^+$ $CD4^+$ T cells in cLPs. Plots represent the mean and values ± S.E.M. Unpaired t-test (B). ***P*<0.01, **P*<0.05.
